# Hyperspectral Raman imaging of neuritic plaques and neurofibrillary tangles in brain tissue from Alzheimer’s disease patients

**DOI:** 10.1038/s41598-017-16002-3

**Published:** 2017-11-15

**Authors:** Ralph Michael, Aufried Lenferink, Gijs F. J. M. Vrensen, Ellen Gelpi, Rafael I. Barraquer, Cees Otto

**Affiliations:** 1 Institut Universitari Barraquer, Universitat Autònoma de Barcelona, Barcelona, Spain; 2 University Eye Clinic, Paracelsus Medical University, Salzburg, Austria; 3 Medical Cell BioPhysics, University of Twente, Enschede, The Netherlands; 4 Department of Ophthalmology, Leiden University Medical Center, University of Leiden, Leiden, The Netherlands; 5 Neurological Tissue Bank of the Biobanc-Hospital Clinic-Institut d’Investigacions Biomediques August Pi i Sunyer (IDIBAPS), Barcelona, Spain; 6 Centro de Oftalmología Barraquer, Universitat Internacional de Catalunya, Barcelona, Spain

## Abstract

Neuritic plaques and neurofibrillary tangles are crucial morphological criteria for the definite diagnosis of Alzheimer’s disease. We evaluated 12 unstained frontal cortex and hippocampus samples from 3 brain donors with Alzheimer’s disease and 1 control with hyperspectral Raman microscopy on samples of 30 × 30 µm. Data matrices of 64 × 64 pixels were used to quantify different tissue components including proteins, lipids, water and beta-sheets for imaging at 0.47 µm spatial resolution. Hierarchical cluster analysis was performed to visualize regions with high Raman spectral similarities. The Raman images of proteins, lipids, water and beta-sheets matched with classical brain morphology. Protein content was 2.0 times, the beta-sheet content 5.6 times and Raman broad-band autofluorescence was 2.4 times higher inside the plaques and tangles than in the surrounding tissue. The lipid content was practically equal inside and outside. Broad-band autofluorescence showed some correlation with protein content and a better correlation with beta-sheet content. Hyperspectral Raman imaging combined with hierarchical cluster analysis allows for the identification of neuritic plaques and neurofibrillary tangles in unstained, label-free slices of human Alzheimer’s disease brain tissue. It permits simultaneous quantification and distinction of several tissue components such as proteins, lipids, water and beta-sheets.

## Introduction

The classical neuropathological features of Alzheimer’s disease (AD) include neuronal loss, astrogliosis and microglial activation, and the presence of neuritic plaques and neurofibrillary tangles in the grey matter. While the hippocampus is involved relatively early, cortical brain areas are affected in later disease stages^1^. The pathological accumulation of amyloid-beta (Aβ) in the brain is caused by an the enzymatic cleavage by α, β and γ secretases of Aβ peptides from the amyloid precursor protein (APP), which can be well visualized by immunohistochemistry with antibodies directed to Aβ_1_–_40_ and Aβ_1_–_42_ peptides^2^. These monomeric peptides are normally broken down by the ubiquitin-proteasome pathway or by phagosomes and lysosomes^3^. In old age, however, this clearance is inhibited and the monomeric peptides tend, for unknown reasons, to aggregate to oligomers, currently considered in their lower molecular weight the most toxic species^4^, and eventually to polymers which form amyloid fibrils. These fibrils are molecularly characterized by high levels of regularly aligned β-pleated sheet configurations and form the main components of the neuritic plaques. Tau is a neuronal microtubule-associated protein whose expression is strongly up-regulated during neuritogenesis^5^. Upon aging the originally unfolded random coil tau protein is altered by several processes and forms fibrils with high levels of β-pleated sheets^6^. The tau fibrils, localized in the neuronal cytoplasm as paired helical filaments on ultrastructure, are the main components of the neurofibrillary tangles which, as the neuritic plaques, alter the normal function of the neurons involved. The trans-synaptic spread of abnormally conformed proteins is currently a topic of interest in neurodegenerative disease, including AD, for which tau is considered one of the main proteins to spread from vulnerable neurons by neuronal connectivity to different brain areas^7^.

The improvement of clinical diagnostic tools for *in vivo* imaging of brains using CT, amyloid-PET and (f)MRI, and the analysis of biomarkers in brain fluids has greatly facilitated the *in vivo* diagnosis of AD over the last decades^8^. Nevertheless definite diagnosis of sporadic AD still requires examination of post mortem brain tissue using different staining procedures. The classical AD histochemical procedures: Congo red, thioflavin S and the Bielschowsky or Gallyas silver technique, label both plaques and tangles in the same section. Immuno-histochemical staining protocols for AD use specific antibodies restricted to a single specific protein (Aβ or tau protein) and give different results for neuritic plaques and neurofibrillar tangles. However the long fixation time for proper preservation of the brain as a whole, the time consuming embedding procedures and the complex histochemical and immuno-histochemical staining protocols make the examination of AD pathology a rather time consuming exercise.

Raman spectroscopy has previously been used to study some aspects of Alzheimer’s disease, such as human Aβ peptides aggregated in solution at room temperature^9^; synthetic Aβ fibrils and in isolated human senile plaques^10^; and AD infected brains in rats^11^ with laser spot diameters of 1.5 to 2 µm and without imaging. Fourier transform infrared spectroscopy has been used for spectral imaging of mouse brain tissue from AD models with a resolution of 5.5 µm^12^. Raman spectroscopy with imaging has been used to characterize human brain tissue with a resolution of 25 µm^13^ and rat brain tissue^14^ with a resolution of 3 µm.

Raman spectroscopy is an optical micro-spectroscopic method, where the sensitive and precise acquisition of spatial- and frequency-resolved light scattering allows identification of groups of macromolecules with identical structural properties. We are suggesting to use hyperspectral Raman imaging of brain tissue at high spatial and frequency resolution, where hyperspectral means that the spectral information (−40 to 3650 cm^−1^) is acquired at every pixel of the scan area. The information in the Raman spectra can be used for the estimation of relative amounts of tissue components as β-sheets, proteins, lipids and water, which can all be imaged simultaneously and used for numerical comparative analysis. An optical resolution of 0.39 µm implemented in the Raman microscope matches the scale of tissue components such as plaques and tangles, which is important to appropriately monitor changes in the molecular content of pathological structures^15,16^. An unmistaken advantage of the hyperspectral Raman imaging approach is that it is a label-free technique, which can be performed on slightly fixed biopsy tissue slices or on cryo-sections of unfixed brain tissue without staining. This greatly reduces the time of AD pathology diagnosis. Raman spectra of all data reported here are acquired at 20 Hz, which corresponds to a pixel dwell time of 0.05 s.

In the present study we have explored the analytical potential of hyperspectral Raman imaging for the identification of plaques and tangles in paraformaldehyde fixed unstained slices from hippocampus and frontal cortex of neuropathological confirmed human AD brain donors.

## Results

### Comparison with histology

**Figure 1:**
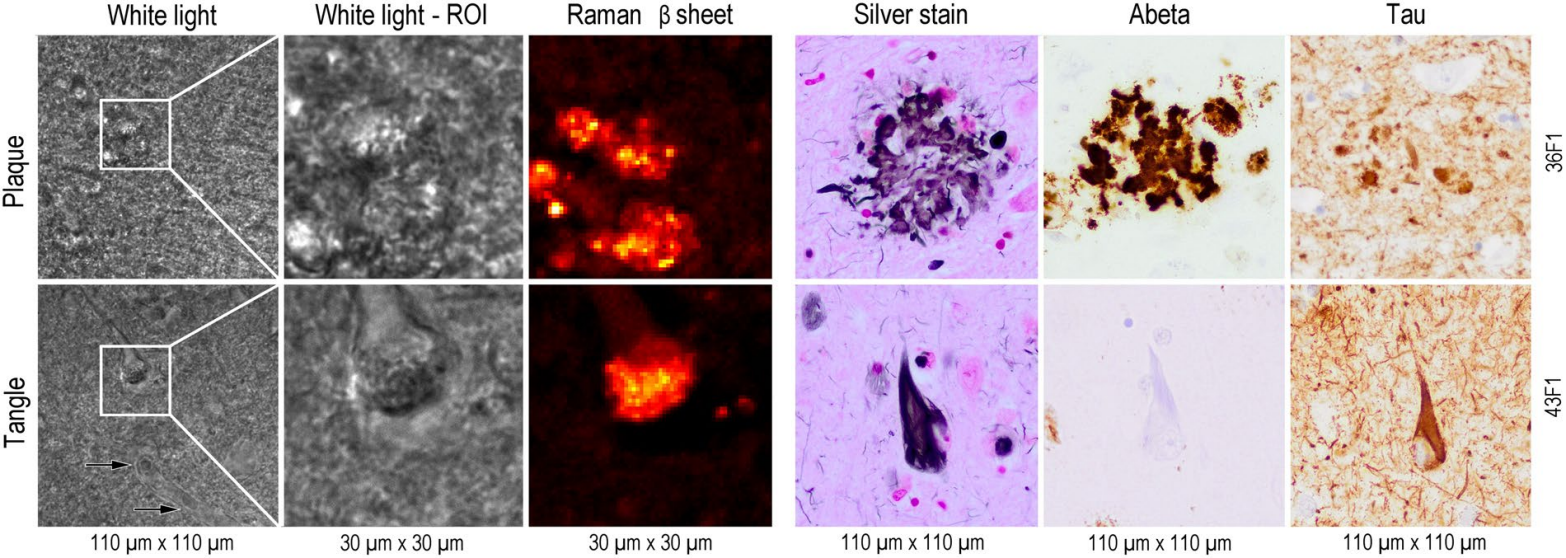
Comparison of the Raman microscopy results with conventionally stained brain sections. White light image from Raman microscope with boxed area indicating the Raman scan region of interest (ROI). Unstained 50 µm thick sections were used for Raman imaging (left panel) and 5 to 8 µm thick sections from an adjacent tissue block were used for conventional histology (right panel). Corresponding Raman intensity image for β-sheets distribution is given next to it. Conventional brain histopathology from the same samples show a neuritic plaque (upper row) and neurofibrillary tangle (lower row), with silver impregnation (Silver Stain) and immunohistochemistry for amyloid-β (Aβ) and hyperphosphorylated tau protein (Tau). Note the lack of Aβ immunostaining of neurofibrillary tangles. Image size is given below and tissue ID numbers are given on the right.

Twelve slices of hippocampus and frontal cortex from three donors with diagnosis AD and one control donor were evaluated. The white light and bright field video mode of the Raman microscope showed structures with multicentre condensations and flame-shaped structures with a defined border (Fig. 1). In each region of interest (ROI) with an area of 30 µm × 30 µm, 4096 localized Raman spectra were recorded in a 64 × 64 grid pattern with an objective with magnification of 40 and a numerical aperture of 0.95.

The first local distribution of Raman scattering intensity was calculated for β-sheet secondary structure (1649–1698 cm^−1^) secondary structure is considered a hallmark of protein aggregation^17^ and folding associated with plaque and tangle formation (Fig. 1). The results were compared with conventional AD staining procedures. Gallyas silver impregnation method labelled both plaques and tangles. Mature plaques were characterized by a condensed centre or by multiple spots which appeared as bulbous bodies without defined borders. The core of neuritic plaques was immuno-positive for Aβ peptide (clone 6 F/3D) while the intermingled neuritic component was immuno-positive for hyperphosphorylated tau (clone AT8) (Fig. 1). In contrast, neurofibrillary tangles had a flame shape or drop-like morphology with a well-defined border, and were immuno-positive for hyperphosphorylated tau, but not for Aβ. The Raman β-sheet intensity corresponded very well with images from conventional histology for AD (Fig. 1).

### Tissue component distribution

**Figure 2:**
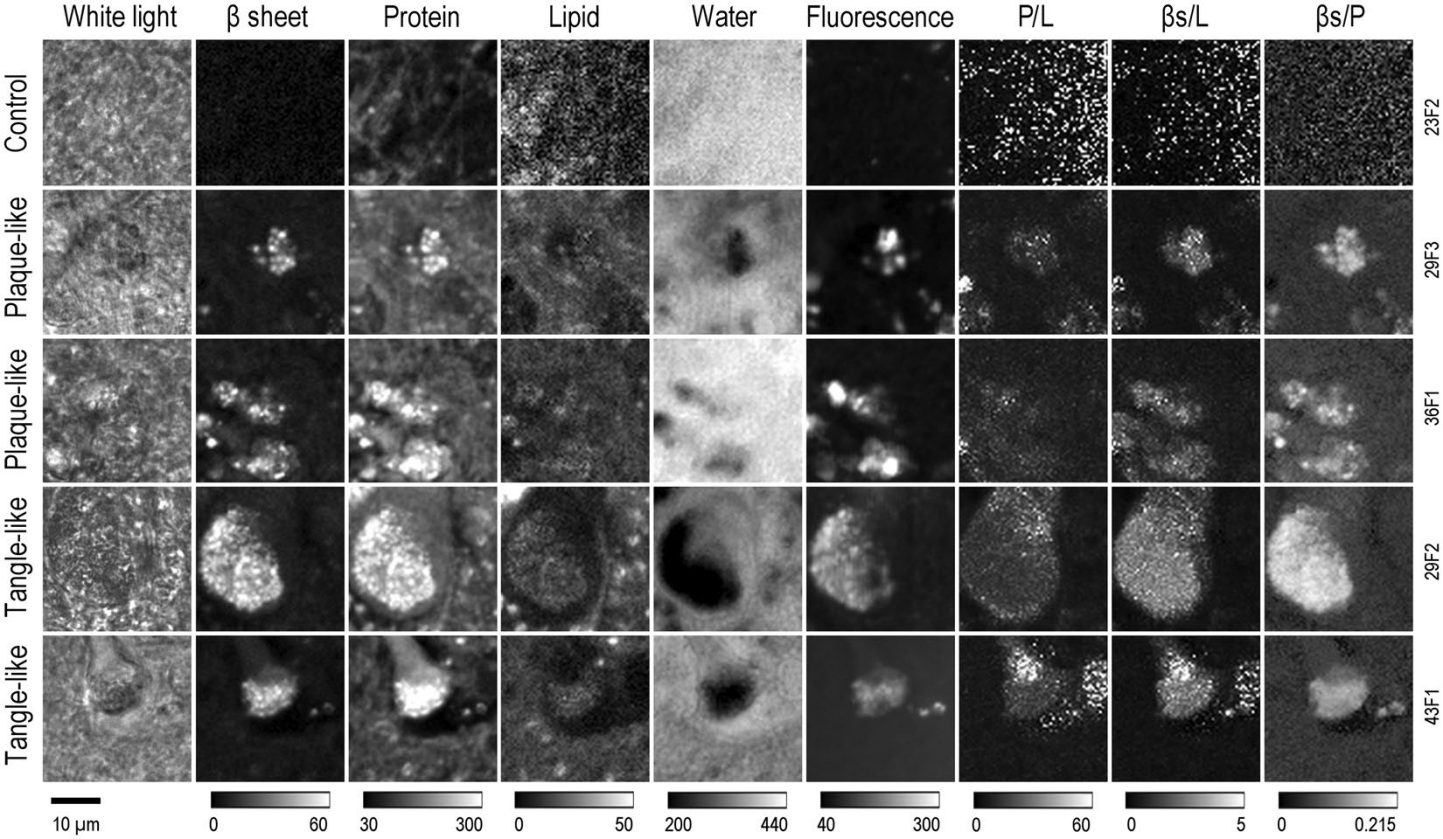
White light images and spatial intensity distributions of Raman signals for β-sheets, proteins, lipids, water, broad-band autofluorescence and protein-to-lipid ratio, β-sheet-to lipid ratio and β-sheets-to-protein ratio. First row shows a control sample, below two samples with plaque-like features and two samples with tangle like features. Grey scale bar below shows the range of the Raman intensity displayed. Image resolution is 64 × 64 pixels showing a tissue area of 30 × 30 µm. Tissue ID numbers are given on the right.

Subsequently, the spatial distribution of more tissue components were calculated using our Raman spectra; proteins (2860–2980 cm^−1^), lipids (2830–2860 cm^−1^) and water (3088–3648 cm^−1^). We also quantified a broad-band autofluorescence which was present as a baseline in the Raman spectra (see below: Cluster analysis). Furthermore, the Raman ratio images were calculated for the protein-to-lipid-, β-sheet-to-lipid- and β-sheet-to-protein-ratios (Fig. 2).

Control regions from a non-AD brain showed low signals of β-sheet protein secondary structure corresponding to a non-pathologic “normal” distribution of β-sheet, protein, lipid and water (Fig. 2). In contrast, plaque-like and tangle-like features in tissue from AD donors showed intense Raman scattering from local accumulation of β-sheet secondary structure and proteins and minor lipid changes. Locations with high protein and high β-sheet showed lower intensities for water. Lipid was reduced just outside of the tangle-like features. The distribution of β-sheet secondary structure, protein and lipid revealed a granular structure inside the feature. However, the respective ratios showed a homogeneous distribution (Fig. 2).

**Figure 3:**
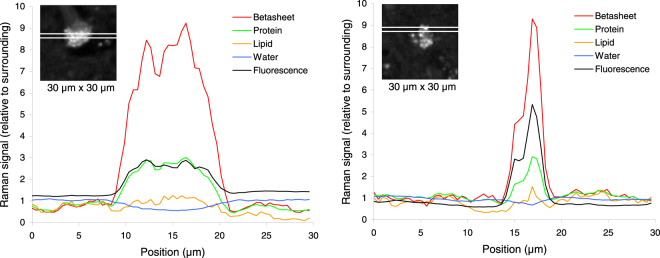
Intensity profiles of different tissue components from two samples shown in Fig. 1 (43F1 and 29F3). The results are given relative to the overall mean of the surrounding tissue from all 12 samples. Insert shows the β-sheet intensity image from Fig. 2 including the strip used for plotting the horizontal profile, which goes 64 pixels (30 µm) across the sample and is averaged over 3 pixels (1.4 µm) vertically.

To further detail the co-localization of the changes in tissue components, intensity profiles were plotted for two samples (Fig. 3). The signal for β sheet secondary structure rapidly increases from the surrounding tissue to inside the feature, reaching local maxima of up to 10 times as compared to the surrounding tissue. Protein and broad-band autofluorescence content increased as well, but lipid content showed only minor variations and water was slightly decreased inside the feature, corroborating the information from Fig. 2. Quantitative tissue component analysis of all 12 samples together, including 49152 Raman spectra (12 × 64 × 64), revealed that the protein content was 2.0 times and the protein β-sheet content 5.6 times higher inside than outside the features (Table 1). Water content inside the features was 0.8 times the amount of water outside. The lipid content was practically equal inside and outside the feature. In many cases, the feature was surrounded by a low lipid content border. The broad-band autofluorescence was 2.4 time higher inside the features.

**Table 1:** Raman intensities in photons per wavenumber for β-sheets, proteins, lipids, water, broad-band autofluorescence and protein-to-lipid ratio, β-sheet-to lipid ratio and β-sheets-to-protein ratio.

	β-sheets	Proteins	Lipids	Water	Fluorescence	Proteins by Lipids	β-sheets by Lipids	β-sheets by Proteins
Surrounding (mean +/− SD)	5.7 ± 2.9	116 ± 34	15.9 ± 8.5	336 ± 55	69 ± 19	10.5 ± 16.3	0.53 ± 0.95	0.049 ± 0.019
Feature (mean +/− SD)	31.9 ± 12.9	232 ± 51	16.6 ± 6.1	272 ± 61	162 ± 58	15.9 ± 11.8	2.12 ± 1.37	0.134 ± 0.031
Feature/Surrounding ratio	5.6	2.0	1.0	0.8	2.4			

Calculations based on all 12 samples and data separated for feature and surrounding tissue according to Supplementary Figure S3.

**Figure 4:**
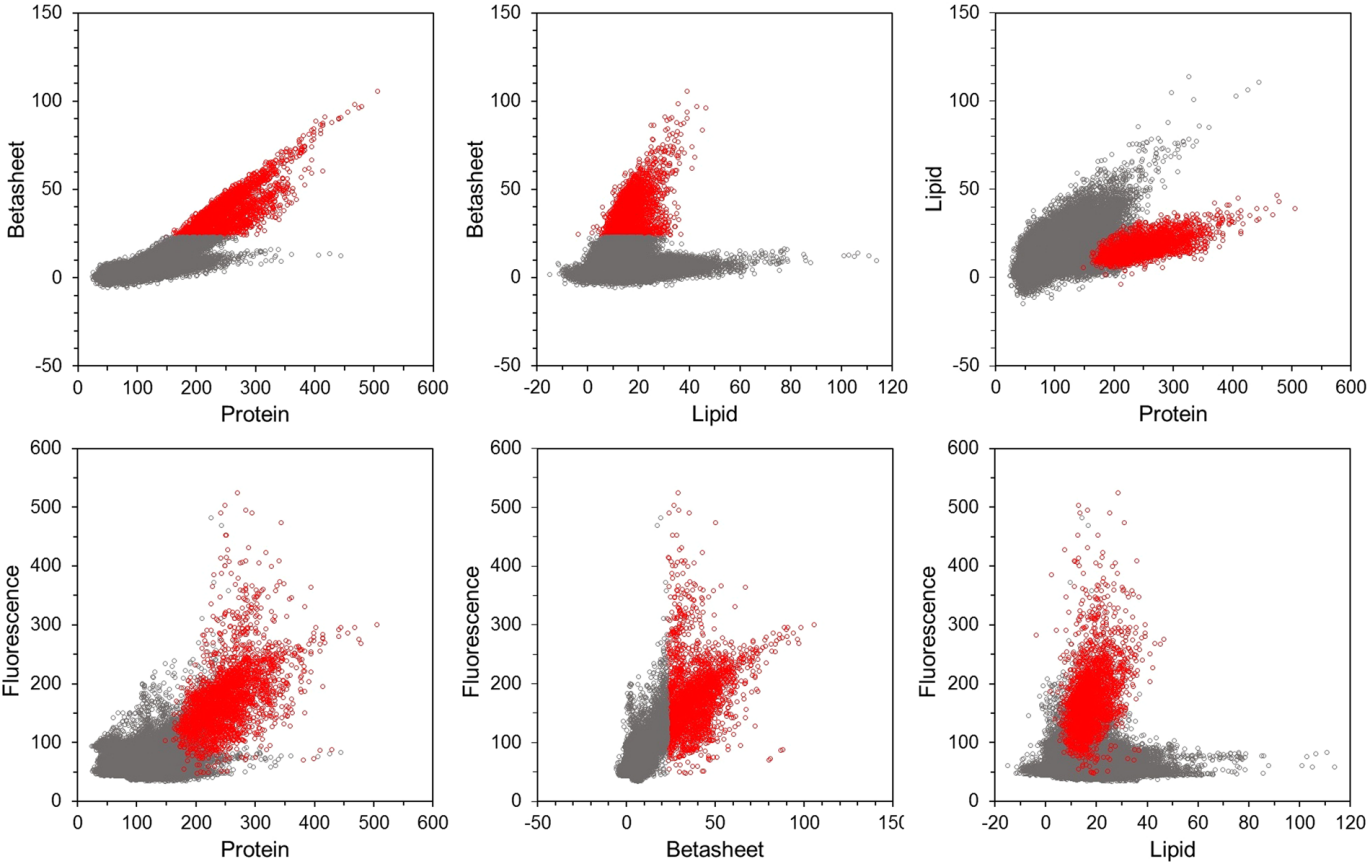
Raman scatter plots showing the spatial correlation of proteins, β-sheets, lipids and autofluorescence. Data from all 12 samples with 4096 data points each, merged and separated for feature (red) and surrounding tissue (grey). Each of the 49152 dots in each graph represent the intensity of the specific Raman intensity indicated along the abscissa and the ordinate.

As expected there was a strong correlation between β-sheet and protein intensities inside the features (R^2^ = 0.67) (Fig. 4). β-sheet intensity is somewhat increasing in the surrounding tissue, and at a certain protein concentration (inside the feature) β-sheets increase linearly with protein concentration. There seems to be also an upper limit for this correlation (no data pixels above a certain protein to β-sheet ratio). β-sheets were correlated with lipids inside the feature (R^2^ = 0.25), but not outside the feature. Lipids were somewhat more correlated with proteins inside the features (R^2^ = 0.42) than outside (R^2^ = 0.31). Although for a certain protein concentration, lipid concentration was lower inside the feature than in the surrounding tissue. Considering features and surrounding together, this autofluorescence showed some correlation with protein content (R^2^ = 0.39) and a better correlation with β-sheet content (R^2^ = 0.55). There was no correlation between autofluorescence and lipids (R^2^ = 0.00).

### Cluster analysis

**Figure 5:**
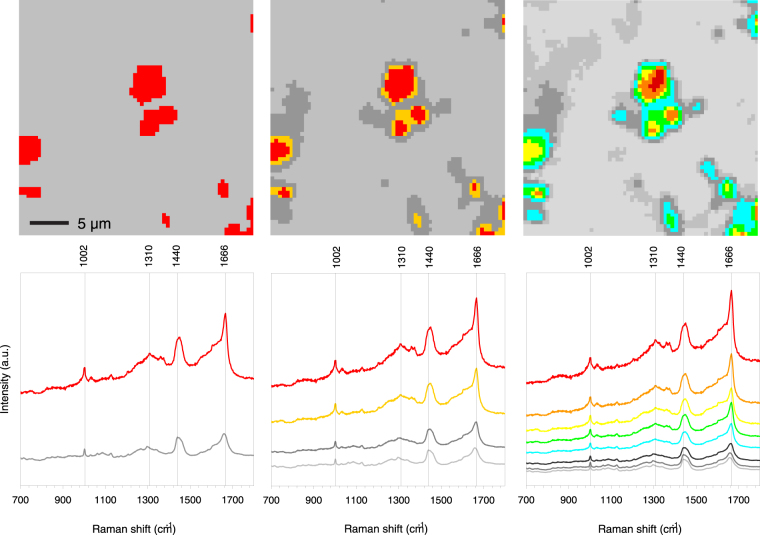
Hierarchical 2, 4 and 8 cluster analysis in the fingerprint Raman shift region (700–1800 cm^−1^) of one tissue sample (29F3). A colour coded image of the Raman scan area region of interest (ROI) is given above each Raman spectra diagram. The colours were assigned from the cluster analysis, which grouped all 4096 local spectra into 2, 4 or 8 groups according to similarities. Therefore, the colours reflect local differences in Raman spectra across the region of interest. For instance, the red Raman spectra is found at locations coloured in red, the grey Raman spectra is found at locations coloured in grey, and so forth. The grey spectra represent the surrounding tissue and the other colours the feature. Image size 30 × 30 µm.

The univariate hyperspectral Raman image analysis requires user input for the selection of the important spectral regions. In medical applications it is of utmost importance to use unbiased and unsupervised data analysis. We have therefore applied various degrees of hierarchical cluster analysis to investigate the detectability of features associated with pathology (Fig. 5). Cluster analysis dissected the data set in 2, 4 and 8 clusters based on the spectral region from 1100 to 1750 cm^−1^ , a spectral range easily encompassed by most Raman imaging instrumentation. Already at the lowest level of cluster analysis the feature recognition was apparent, while higher levels of cluster analysis, i.e. to 4- and 8-cluster levels, introduces a more refined view on the spread of pathology throughout the tissue.

The 2-level cluster analysis clearly points out that the prominent spectral differences were in broad-band autofluorescence, the pronounced band at 1666 cm^−1^ (β-sheet) and a broadening of the 1002 cm^−1^ band of phenylalanine. The 4 and 8 cluster analyses highlighted the spectral differences even more. Progressive levels of cluster analysis brings out more detailed changes in the spectra that suggests that a gradual change in chemistry occurred starting from the local core of a feature and radiating outwards towards the surrounding brain tissue. Figure 5 is an example of an area where multiple feature cores can be distinguished. The core of the feature is dressed with the colour “red” and the colour-corresponding cluster spectrum is shown in the lower graph. The next cluster from the core is dressed with the colour “orange”. This sequence of chemical changes with respect to the light grey surrounding tissue can also be observed in other cores within this ROI. We conclude that the chemical information, encoded in the cluster colours, follows a spatial organization, which is reproducible from one core to another throughout the 8 cluster image (Fig. 5). Such results on individual ROI’s led to the hypothesis that if chemical changes are so remarkably preserved, this could also be the case when multiple ROI’s are assembled together and analysed as one single data set by multivariate procedures, more precisely principal component analysis followed by hierarchical cluster analysis.

**Figure 6:**
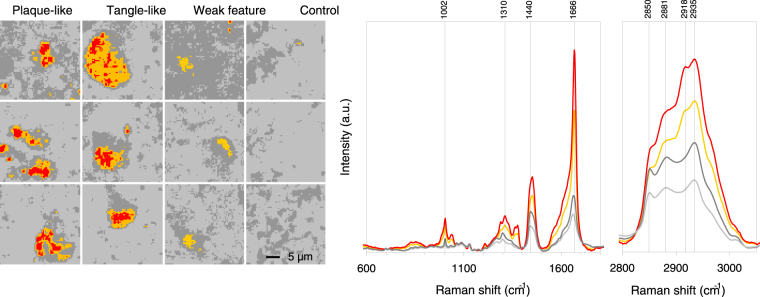
Combined hierarchical 4 cluster analysis of all 12 samples together. On the left false colour representation of all samples with an individual resolution of 64 × 64 pixels showing a tissue area of 30 × 30 µm for each sample. Grey colours correspond to the surrounding tissue and the orange and red colours to the feature body and core. The spectra are given with the corresponding colours for the fingerprint region (700–1800 cm^−1^) and the high frequency region (2800–3500 cm^−1^), representing the mean results from 49152 spectra (64 × 64 pixels from 12 samples). Tissue ID numbers from left to right and top to bottom are: 29F3, 29F2, 29F1, 23F1, 36F1, 36H3, 36H1, 23F2, 29F4, 43F1, 36H2, 23H1. The indicated band positions are assigned to phenylalanine (1002 cm^−1^), CH2/CH3-deformation mode^13^ (1310 cm^−1^), -CH2-bending mode^28^ (1440 cm^−1^), protein β-sheet^17^ (1666 cm^−1^), -CH2-symmetric stretching mode^29^ (2850 cm^−1^), the -CH2-antisymmetric stretching mode^29^ (2881 cm^−1^), Fermi resonance -CH2- + antisymmetric CH bending mode^29^ (2918 cm^−1^), lipid and protein -CH3 symmetric stretching mode^28^ (2935 cm^−1^).

To test this hypothesis, we assembled all 49152 Raman spectra from the 12 samples, removed the broad-band autofluorescence and performed a hierarchical cluster analysis on the combined dataset. The results of the cluster analysis is given in Fig. 6, presenting 4 cluster spectra. We assigned two shades of grey to the spectra with the lowest bands at 1666 cm^−1^ (β-sheet) and red and orange to the spectra with the highest intensities of this band. Comparison with the cluster images revealed that both grey spectra corresponded to the surrounding tissue, the red spectra to the feature cores and orange to the feature body (Fig. 6). We conclude that the chemical changes in 12 different areas of brain sample from 3 donors with described AD pathology are reproduced throughout the combined hyperspectral Raman imaging dataset and that the chemical changes are therefore a robust marker for Alzheimer disease pathology.

## Discussion

Conventional neuropathological examination, still crucial for AD diagnosis, served as a reference in the present study. In the hyperspectral Raman images multicentre condensations with loose, ill-defined borders as well as flame-shaped structures with defined borders were found. Both had a comparable size and morphology as conventionally stained AD tissue (Fig. 1). Because the centre of these features was characterized by high amounts of β-sheets, a main and common molecular characteristic of Aβ and tau protein^2,18^, we conclude that we are indeed able to locate and image plaques and tangles using label-free, high spatial and spectral resolution spontaneous Raman spectroscopy.

Neuritic plaques and neurofibrillary tangles contain large amounts of aggregated protein. Quantitative tissue component analysis revealed two times more proteins and five times more β-sheets inside the plaque- and tangle-like features as compared to the surrounding tissue (Fig. 2). This indicates that both protein aggregation and conversion of unordered protein secondary structure to β-sheet secondary structure occur. This is in agreement with the generally accepted view on amyloidosis^18^. Similar levels of lipids in- and outside the feature (Fig. 2) confirmed an earlier finding in AD models in mouse brain tissue^12^. Raman images show a similar granular distribution for both the β-sheet signal centered at 1666 cm^−1^ and the protein signal centered at 2935 cm^−1^. This positive correlation of β-sheet secondary structure with areas with a dense packing of proteins inside the features suggests that above a certain protein level specific folding of proteins into β-sheet secondary structures occurs (Fig. 4).

**Figure 7:**
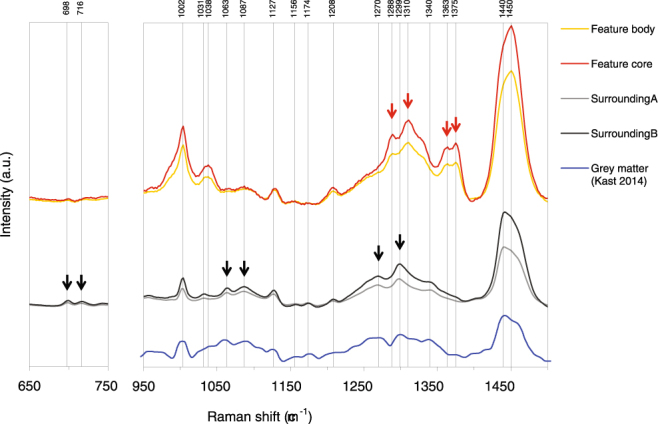
Detailed view of the Raman spectral regions between 650 and 750 cm^−1^ and between fingerprint region between 950 and 1500 cm^−1^ of the combined hierarchical 4 cluster analysis shown in Fig. 6. The spectra from the feature body and core (orange and red) are shifted up to allow better comparison with the spectra from the surrounding tissue (grey). The main changes are indicated with arrows, black arrows indicate Raman bands disappearing in the feature regions and red arrows indicate Raman bands appearing in the features. A Raman spectra from the grey matter of a normal human brain^13^ is shown below for comparison.

The position of the Amide I band maximum found at 1666 cm^−1^ in our human brain tissue (Figs 5, 6 and 7) is in good agreement with the Raman spectrum of α-synuclein in β-sheet structure in aqueous solution (Supplementary Fig. S1). Our assignment of the Raman band between 1649 and 1698 cm^−1^ (maximum at 1666 cm^−1^) in the Amide I envelop as β-sheet secondary structure is also in good agreement with the literature for Raman spectroscopy^17,19,20^. More details and references for the assignment of the Amide I proteins for Raman spectroscopy and compared with assignments for infrared absorption spectroscopy is given in Supplementary Table 1.

Hierarchical cluster analysis of the Raman spectra suggest a progression from a classical protein-lipid spectrum in the surrounding tissue towards more compacted, multi-centered protein condensations into the features (Fig. 6). It appeared as an evolution of a conservative, classical protein-lipid spectrum with normal (aromatic) amino acid contributions and normal lipid contributions to pathological tissue with intense β-sheet bands on top of a pronounced broad-band auto-fluorescence. The cluster analysis further show that the contribution of phosphatidylcholine (716 cm^−1^) and cholesterol (698 cm^−1^) is not evident in the feature areas (Fig. 7), while the presence is evident in the surrounding tissue.

Potentially, the emission of fluorescence may contribute to the Raman spectrum. This is indeed the case in the present results (Figs 2 and 5), where intense Raman signal and a relatively weak near infrared fluorescence emission signal overlap on the CCD detector. This broad-band fluorescence emission correlated with the β-sheet and protein distribution and hence with the plaques and tangles (Figs 2 and 5). The fluorescent emission intensity was more than two times higher inside the feature than outside. The origin of the broad-band autofluorescent background is not known, but it might be due to protein degradation, as we also notice that the totally symmetric ring stretching vibration of phenylalanine at 1002 cm^−1^ decreases in amplitude concomitant with a change in band-shape. Interestingly, such a pronounced fluorescence emission has been mentioned earlier in human brain tissue from AD donors and it has been suggested to be used for diagnosing AD in living patients^21^. However, the observation was not further explored. We now add a clear correspondence between the intense β-sheet Raman signals and the near-infrared fluorescence emission, suggesting a set of potentially diagnostically relevant optical signals for AD detection in brain tissue. Having established this relationship, it is reasonable to suggest that especially the amplitude of the fluorescence emission coincident with the Raman spectrum between 645 nm and 848 nm could be useful for diagnostic purposes. The origin of the fluorescence emission is not clear. Although lipofuscin has been proposed^21^ as a candidate near infrared fluorescence emitter, and it is especially abundant in neurons of aged and AD brains we consider it is less likely given the decrease in amplitude of lipofuscin fluorescence from 700 nm towards the near infrared, opposite to what we observe. We suggest that the near infrared fluorescence emission is connected to dense protein aggregates mixed with lipids and other metabolic products obtained after oxidation.

The spectra of the surrounding tissue (Figs 6 and 7; light grey and dark grey) were dominated by contributions of both protein and lipids. The protein contribution was characterized by the well-known resonances of aromatic amino acids (1002 cm^−1^, 1031 cm^−1^ phenylalanine, 824 cm^−1^, 850 cm^−1^ tyrosine, 1208 cm^−1^ tyrosine and phenylalanine) together with amide III mode (1230–1310 cm^−1^) and amide I mode (1630–1680 cm^−1^), which are typical for a mixed secondary structure. The phenylalanine band (1002 cm^−1^) had a band width in agreement with phenylalanine in standard protein Raman spectra. The classical protein spectrum was overlapping with cholesterol and phosphatidylcholine lipid bands with maxima at 698 cm^−1^, 716 cm^−1^, 1063 cm^−1^, 1087 cm^−1^, 1127 cm^−1^ ^14^ and 1658 cm^−1^. The latter band is due to the C=C stretching mode of unsaturated lipids, a band which is overlapping with the amide I mode of proteins (1630–1680 cm^−1^), but clearly distinct from the position of the intense β-sheet band at 1666 cm^−1^ that was observed in the spectra of the feature. The band intensity of the carbon-hydrogen bond stretching vibrations connected to unsaturated C=C bonds occurs at 3022 cm^−1^ can be observed in Fig. 6. The band does not increase in intensity in going from surrounding tissue (light grey) towards the tissue in features (red). It is therefore reasonable to state that the sharp rise in β-sheet is not due to an increase in unsaturated –C=C- bond. The Raman spectrum of the surrounding tissue is characteristic of the expected lipid to protein ratio. The maximum of the 1430–1470 cm^−1^ band at 1440 cm^−1^ is common in spectra from brain tissue and typical for the presence of cholesterol and lipids. The spectra of the surrounding brain tissue correspond with earlier Raman results on normal human brain tissue from surgical biopsies (grey matter)^13^ and normal rat brain tissue^14^ (Figs 6 and 7).

The feature spectra (Figs 6 and 7; yellow and red) were non-classical spectra dominated by an intense β-sheet band (1666 cm^−1^) and a shift in the maximum of the 1430–1470 cm^−1^ band from 1440 cm^−1^ to 1450 cm^−1^ typical for pure protein spectra^15^. In the feature spectrum the cholesterol (698 cm^−1^) and phosphatidylcholine (716 cm^−1^) peaks, the lipid peaks at 1063 cm^−1^ and 1087 cm^−1^ and the amide III peaks at 1208 cm^−1^ and 1299 cm^−1^ are absent or greatly reduced. The 1288 cm^−1^, 1299 cm^−1^,1363 cm^−1^ and 1375 cm^−1^ peaks are much more pronounced in the feature than in the surroundings. They can be ascribed to cytosine, CH_3_/CH_2_ bending of lipids, guanine, and the thymine, adenine and guanine breathing modes of DNA/RNA bases respectively^22^. Although bands at 1288 cm^−1^ and 1363 cm^−1^ and 1375 cm^−1^ correspond in position with those of nucleobases of DNA many other important DNA bands are missing (680 cm^−1^, 730/750 cm^−1^, 790 cm^−1^, 1095 cm^−1^, 1480 cm^−1^ and 1578/1580 cm^−1^).

## Conclusion

The present study showed that hyperspectral Raman imaging combined with hierarchical cluster analysis, is able to identify neuritic plaques and neurofibrillary tangles in unstained, label-free slices of human Alzheimer’s disease brain tissue. The Raman signals of plaques and tangles strikingly differ from the surrounding tissue. A spatial resolution of 0.47 µm and quantification of main tissue components such as proteins, lipids, water and β-sheets conformation allow their co-localization and evaluation of the molecular changes during plaque and tangle formation. Our fast hyperspectral Raman imaging with a dwell time of 50 ms/pixel can be considered in general as a valuable tool for identification of lesions in pathological tissues characterized by storage of macromolecules with specific molecular characteristics. The study also revealed that fluorescence emission of unknown endogenous fluorophores correlates with the observable changes at the locations of the plaques and tangles.

## Materials and Methods

### Brain donor information

Brain tissue from four donors was provided by the Neurological Tissue Bank of the Biobank-Hospital Clinic-IDIBAPS, Barcelona, Spain (NTB-IDIBAPS). Written informed consent for removal of the brain for diagnostic and research purposes was obtained from patients and/or relatives. The research was carried out in accordance with the tenets of the Declaration of Helsinki on research involving human subjects. The experimental protocol was approved by the Ethical Committee for Clinical Research of the Centro de Oftalmología Barraquer. Alzheimer’s disease staging was classified according to the National Institute on Aging and the Alzheimer’s Association guidelines “ABC” score for Alzheimer’s disease neuro-pathologic change^23^.

We evaluated a total of 12 frontal cortex and hippocampus samples from 3 brain donors with Alzheimer’s disease and 1 control donor. Brain tissue from the frontal cortex (grey matter; Brodman area BA 9/10) and the hippocampus (CA1 region) were studied. Tissue samples were coded as follows: first two digits = brain donor ID; letter: F = frontal cortex, H = hippocampus; last digit: running number for different scan areas.

Brain tissue from an 82 year old female with a low ABC score (A1, B1, and C0) served as control. The tissue showed argyrophilic grain disease, vascular-atherosclerotic encephalopathy and multiple old infarcts. Two areas of the frontal cortex (23F1, 23F2) and one area of the hippocampus (23H1) were studied. The three other donors had high ABC scores for Alzheimer’s disease: a 79 year old female (A3, B3, C3) with additional neocortical Lewy bodies, small vessel disease with old infarcts - 4 areas of the frontal cortex were studied (29F1, 29F2, 29F3, 29F4); a 91 year old female (A3, B3, C3) with additional small vessel disease, limbic TDP43 protein aggregates - 1 area of the frontal cortex (36F1) and 3 areas of the hippocampus (36H1, 36H2, 36H3) were studied; a 63 year old female (A3, B3, C3) with additional neocortical Lewy bodies, acute cerebellar infarct - 1 area of the frontal cortex (43F1) was studied.

### Tissue preparation

For conventional histology, the tissue was fixed in 4% formaldehyde solution for 3–4 weeks, embedded in paraffin and cut in 8 µm thick sections for silver stains and 5 µm for immuno-histochemical techniques. After de-paraffination, sections were stained with Gallyas silver impregnation method. Immunohistochemistry for amyloid-β and hyperphosphorylated tau was performed on an automated immuno-stainer (DAKO Autostainer plus, Glostrup, Denmark) using the following mouse monoclonal primary antibodies: anti- Aβ: clone 6 F/3D (Dako, Glostrup, Denmark; dilution 1/400) and anti- hyperphosphorylated tau protein: clone AT8 (Thermo scientific, Rockford, IL, USA; dilution 1:200). Immunoreaction was visualized using the Envision+ system peroxidase procedures (DAKO) with diaminobenzidine as chromogen.

For label-free Raman spectroscopy, an adjacent tissue block from the same area was fixed in 4% paraformaldehyde in 0.08 M cacodylate buffer (pH 7.3) for maximal one week and then stored in 0.2% paraformaldehyde in 0.08 M cacodylate buffer (pH 7.3) for 5 weeks. It was cut with a vibratome (Leica 1000 S) in slices of 50 µm thickness and stored in PBS for Raman analysis, which was carried out within one week after slicing.

### Raman spectroscopy

Non-resonant Raman spectroscopy and imaging experiments were performed on a previously described laser-scanning confocal Raman microspectrometer^24^. The system consist of a Krypton laser (Innova 90-K; Coherent, Santa Clara, CA, λ_exc_647.1 nm), an upright microscope BX41 from Olympus with an objective Olympus, Plan Apochomat, 40x, 0.95NA, cover slip corrected for illumination of the sample as well as for collection of Raman scattered photons. The home-built spectrograph was optimized for broadband (−40 to 3650 cm^−1^) high-wave number-resolution (1.85 to 2.85 cm^−1^/pixel) Raman micro-spectroscopy. Imaging experiments were performed by raster-scanning the laser beam over a region of interest with a step size of approx. 0.47 µm (30 × 30 µm containing 64 × 64 spectra) with a dwell time per pixel of 50 ms. The tissue samples were scanned between 5 to 10 µm below the section surface and the z-resolution was 2 µm. A full image of each area was thus acquired in ~205 seconds with a laser power of 35 mW. The measured Raman data were corrected by standard procedures for cosmic ray removal and of pixel-to-pixel variation versus a calibrated light source. Noise in the resulting 3D (spatial × spatial × spectral dimension) data matrix of 4096 spectra times 1600 frequencies was reduced by singular value decomposition^25^.

The raw Raman spectroscopic data from individual 64 × 64 pixel datasets were converted to real data by the following steps: 1) Cosmic ray removal. Occasionally a cosmic ray hits the CCD detector surface releasing electrons from the active area into the well-capacitor. These “cosmic rays” are characteristically intense, local at a limited number of pixels and not reproducible from spectrum to spectrum. On the basis of these criteria the cosmic rays were removed from spectra in the dataset and replaced by number matching the numbers in adjacent spectra. 2) Pixel-to-wavenumber calibration. The 1600 pixels along the spectral axis on the CCD camera were assigned to wavenumbers after recording two calibration spectra. The first spectrum is from an Ar-Hg lamp (Cal-2000, Ocean Optics Mikropack, Germany) with well-known atomic emission band positions in nanometres, independent of the Raman laser wavelength. The second spectrum is the Raman spectrum of toluene, which has well-established band positions (McCreery research group, University of Alberta, http://www.chem.ualberta.ca/~mccreery/ramanmaterials.html) in wavenumber (cm^−1^). The actual wavelength associated with each wavenumber does depend on the excitation wavelength in nanometre, which coincides with 0 cm^−1^ in the Raman spectrum. This position in pixels was introduced also in the pixel-to-wavenumber conversion of the spectral axis. 3) White light correction. Since the pixels of the CCD camera do not have an equal response and the transmission of the complete microscope, from the sample to the detector plane, is not equal as well as that the quantum yield of the detector is not equal for all incident wavelengths, it is necessary to correct for these variations with the help of a calibrated “white light” emission lamp (Avalight-Hal, Avantes, The Netherlands). After this spectral flattening procedure, the amplitude is converted to photons, and all pixels are comparable with each other. The Raman intensity of toluene was used to acquire data at similar alignment precision on a day-to-day basis.

### Tissue component analysis

Raman intensities for specific bands representing the following tissue components were calculated: β-sheets (1649–1698 cm^−1^), proteins (2860–2980 cm^−1^), lipids (2830–2860 cm^−1^) and water (3088–3648 cm^−1^). Raman intensity for each band was corrected by the local baseline on the basis of two intervals left and/or right of the band (Supplementary Fig. S2). To quantify the broad-band autofluorescent background, a Raman band without peaks (1800–2200 cm^−1^, the so-called silent region) was selected and not corrected for baseline. Raman intensity for each band was integrated and its value divided by the wavenumber bandwidth, giving an average in photons per wavenumber. This whole procedure was done for all 4096 spectra individually within the data matrix for each of the 12 tissue samples. Before calculating any tissue component ratio, all negative and zero values were converted to +0.5 (photons) to avoid extreme and negative ratios in case of noise dominated signal. Ratios were calculated by dividing two data sets point by point.

The data matrices of all 12 tissue samples were combined and converted into a 32-bit TIFF image for each tissue component or ratio. Based on the β-sheet data set, an image mask was created to separate the areas of the features from that of the surrounding tissue (Supplementary Fig. S3). This was done by thresholding using the Maximum Entropy method^26^ in ImageJ. This mask was applied to all other tissue components and ratios to calculate the mean and standard deviation of all 12 samples together (Table 1). For visualization, extreme values were clipped by applying the same minimum and maximum values for the combined 32-bit TIFF image presented in grey scale. In this way, the same grey scale value represented the same amount of the tissue component across all 12 samples (5 samples are shown in Fig. 2).

Correlation analysis was done to compare the local Raman intensities for proteins, β-sheets, lipids and broad-band autofluorescence inside the 12 tissue samples (ROI). The same data matrices described above with separation for features and surrounding tissue were used. In this way, the correlation analysis is based on 49152 Raman spectra (12 samples times 64 × 64 data points per sample). Each data point in the scatter plot (Fig. 4) shows the Raman intensity for two tissue components (x and y data) at the same pixel location inside the ROI.

### Cluster analysis

Hierarchical cluster analysis was performed on Raman imaging data matrices to visualize regions with high Raman spectral similarities within the 30 × 30 µm samples. In the cluster analysis routine scores derived with principal component analysis using the standard matlab function “princomp” from the statistical toolbox of matlab. The principal components were taken as input variables for the cluster analysis routine using the standard matlab function “linkage” also available in the statistical toolbox. As further input in this function, Ward’s agglomerative hierarchical clustering based on squared Euclidean distances was employed to partition Raman data sets into clusters. In the present study we have presented 2, 4 and 8 levels cluster analysis as an adequate means to convey the consistency in the spectral analysis of and around each feature. Hierarchical cluster analysis was applied for each sample individually on the instrument corrected raw data without baseline removal or smoothing (Fig. 5). Prior to the combined cluster analysis of all 12 samples together, the broad-band auto fluorescent baseline signal was removed by a Whittaker smoother^27^. The spectra resulting from this combined cluster analysis were smoothed by a moving average filter with a span of 3 (replacing each data point with the average of the neighbouring data points) (Figs 6 and 7).

### Data Availability

The datasets generated during and/or analysed during the current study are available from the corresponding author on reasonable request.

## Electronic supplementary material

Supplementary material
