# Supplementary material for: Hyperspectral Raman imaging of neuritic plaques and neurofibrillary tangles in brain tissue from Alzheimer’s disease patients
Source: Sci Rep. 2017 Nov 15;7:15603. doi: 10.1038/s41598-017-16002-3 (PMC5688091; doi:10.1038/s41598-017-16002-3)
Supplement: Supplementary file 1 — Supplementary material [file 41598_2017_16002_MOESM1_ESM.pdf]

Hyperspectral Raman imaging of neuritic plaques and neurofibrillary tangles in brain tissue from Alzheimer's disease patients

Ralph Michael[1,2], Aufried Lenferink[3], Gijs F.J.M. Vrensen[4], Ellen Gelpi[5], Rafael I. Barraquer[1,6], Cees Otto[3]

1 Institut Universitari Barraquer, Universitat Autònoma de Barcelona, Barcelona, Spain
2 University Eye Clinic, Paracelsus Medical University, Salzburg, Austria
3 Medical Cell BioPhysics, University of Twente, Enschede, The Netherlands
4 Department of Ophthalmology, Leiden University Medical Center, University of Leiden, The Netherlands
5 Neurological Tissue Bank of the Biobanc-Hospital Clinic-Institut d'Investigacions Biomediques August Pi i Sunyer (IDIBAPS), Barcelona, Spain
6 Centro de Oftalmología Barraquer, Universitat Internacional de Catalunya, Barcelona

Supplementary Table 1

Assignments of the Amide I of proteins in Raman spectroscopy and infrared absorption spectroscopy

Amide I	Raman spectroscopy in [cm^{-1}]	Ref.	Infrared absorption spectroscopy in [cm^{-1}]	Ref.
α-helix	1650-1655<br>1645-1655	1<br>4	1648-1657<br>1650-1657	2, 3<br>5
β or extended structures	1665-1670<br>1668-1673 strong<br>1669	1<br>6<br>8	1623-1641 strong<br>1674-1695 weak<br>1628-1634 strong<br>1612-1640 antiparallel beta-sheet<br>1670-1690 weak<br>1626-1640 parallel beta-sheet	2, 3<br>2, 3<br>6<br>5<br>5<br>5
Turn	1662-1672	7	1662-1686<br>1655-1675<br>1680-1696	2, 3<br>5<br>5
Unordered	1639 (max)<br>1638 (broad)	8<br>9	1642-1657<br>1640-1651	2, 3<br>5

For additional information on “turns”, we refer to an extensive review by Vass et. al., added as reference 10 below.

References:
1) N.C. Maiti, M.M. Apetri, M.G. Zagorski, P.R. Carey, V.E. Anderson, (2003) Raman Spectroscopic Characterization of Secondary Structure in Natively Unfolded Proteins: α-Synuclein, *J. Am. Chem. Soc.* 2004, 126, 2399-2408, doi: 10.1021/ja0356176
2) A. Barth, (2007) Infrared spectroscopy of proteins, *Biochimica et Biophysica Acta* 1767, 1073-1101
3) E. Goormaghtigh, V. Cabiaux, J.-M. Ruysschaert, (1994) Determination of soluble and membrane protein structure by Fourier transform infrared spectroscopy: III Assignments and model compounds, *Subcell. Biochem*. Vol. 23, 405-450, eds. H.J. Hilderson and G.B. Ralston
4) Chen, P., et al. Raman signature from brain hippocampus could aid Alzheimer's disease diagnosis. *Appl. Opt.* 48, 4743-4748 (2009)
5) J.T. Pelton and L.R McLean, (2000) Spectroscopic methods for analysis of protein secondary structure, *Anal. Biochemistry* 277, 167-176
6) L. Breydo, D. Kurouski, S. Rasool, S. Milton, J.W. Wu, V.N. Uversky, I.K. Lednev, C.G. Glabe, (2016) Structural differences between amyloid beta oligomers. *Biochemical and biophysical research communications* 477, 700-705
7) H. Ishizaki, P. Balaram, R. Nagaraj, Y.V. Venkatachalapathi, A.T. Tu, (1981) Determination of β-turn conformation by laser Raman spectroscopy. *Biophysical Journal* 36, 509-517
8) T. Lefèvre, M.-E. Rousseau, M. Pézolet, (2007) Protein Secondary Structure and Orientation in silk as revealed by Raman spectromicroscopy. *Biophysical Journal* 92, 2885-2895
9) S.L. Hsu, W.H. Moore, S. Krimm, (1977) Vibrational spectrum of the unordered polypeptide chain: a Raman study of feather keratin. *Biopolymers* 15, 1513-1528
10) E. Vass, M. Hollósi, F. Besson, R. Buchet, (2003) Vibrational spectroscopic detection of beta- and gamma-turns in synthetic and natural peptides and proteins. Chem. Rev. 103, 1917-1954

Supplementary Figure S1

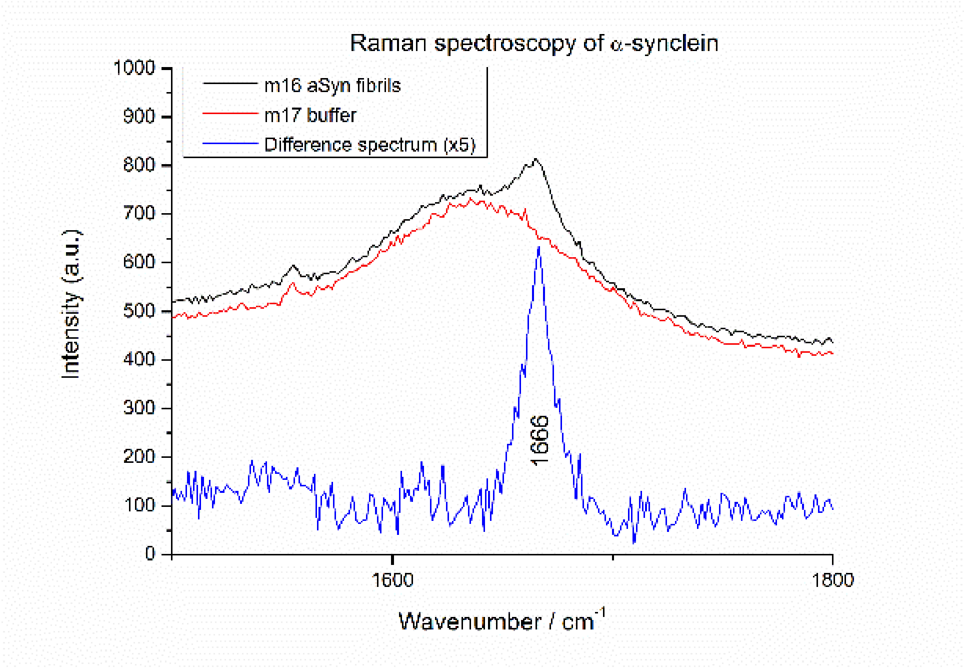

The Raman spectrum of alpha-synuclein in β-sheet structure in aqueous solution.
(Previously unpublished data by ME van Raaij, I. Segers-Nolten, C. Otto, V. Subramaniam).

Supplementary Figure S2

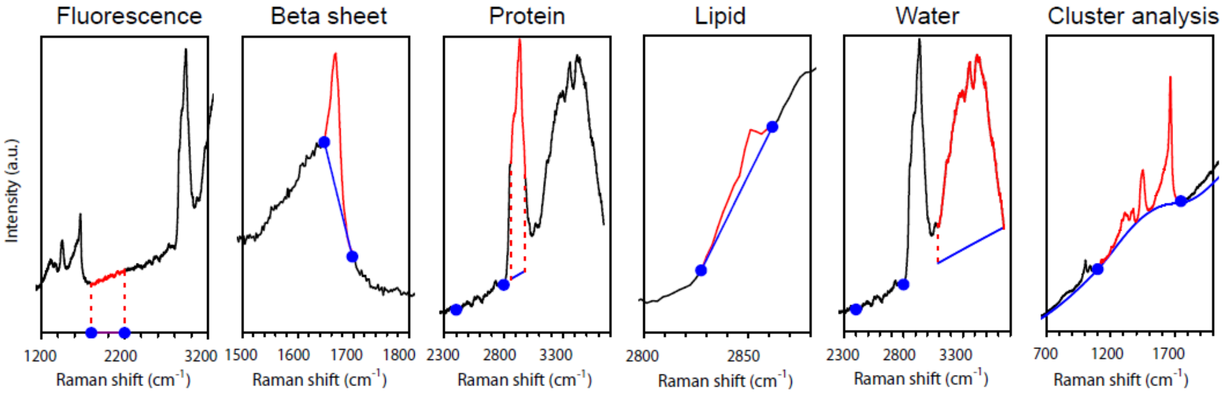

Raman intensity integration for broad-band autofluorescence background (1800-2200 cm-1), β-sheet (1649-1698 cm-1), protein (2860-2980 cm-1), lipid (2830-2860 cm-1) and water (3088-3648 cm-1). The baseline removal before the combined cluster analysis is shown on the right. The spectral band used for integration are shown in red. The blue dots indicate the points used for baseline estimation and the blue lines the estimated baseline which was subtracted from the spectral band intensity. The prominent peaks inside the Raman bands are visible for β-sheet at 1666 cm-1, for protein at 2935 cm-1 and for lipid at 2850 cm-1.

Supplementary Figure S3

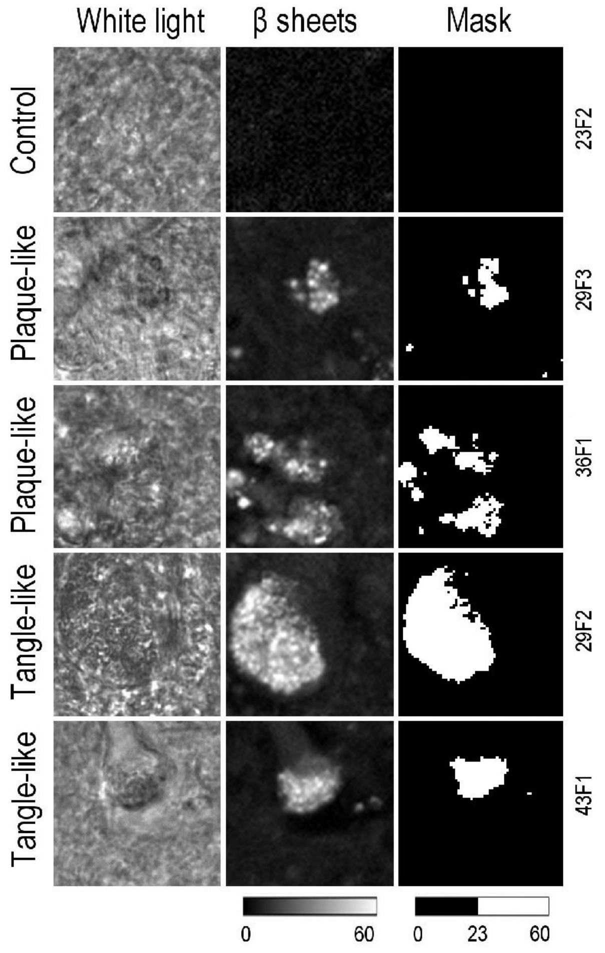

Illustration that shows the creation of the image mask used for separation of the areas of the features from that of the surrounding tissue. Maximum entropy thresholding was applied to the β-sheet intensity results (Fig 2). White light images from the bright field video mode of the Raman microscope are given for reference on the left. Tissue ID numbers are given on the right; image size is of 30 x 30 µm. First row shows a control sample, below two samples with plaque-like features and two samples with tangle-like features. Grey scale bar below show the range of the Raman intensities displayed.
